# Evolution of Prime Editing: Enhancing Efficiency and Expanding Capacity

**DOI:** 10.1002/advs.202521015

**Published:** 2026-02-11

**Authors:** Jihyeon Yu, Ju‐Chan Park, Heesoo Uhm, Yong‐Woo Kim, Hyeon Woo Im, Sangsu Bae

**Affiliations:** ^1^ School of Biomedical Convergence Engineering Pusan National University Yangsan Republic of Korea; ^2^ Genomic Medicine Institute Seoul National University College of Medicine Seoul Republic of Korea; ^3^ Harvard Medical School Boston Massachusetts USA; ^4^ Department of Biomedical Sciences Seoul National University College of Medicine Seoul Republic of Korea; ^5^ Cancer Research Institute Seoul National University College of Medicine Seoul Republic of Korea; ^6^ Institute of Molecular Biology and Genetics Seoul National University Seoul Republic of Korea

**Keywords:** base editing, CRISPR‐Cas, double‐strand breaks, genome engineering, prime editing

## Abstract

Genetic mutations cause approximately 80% of rare human diseases, highlighting the urgent need for precise genome editing. Since clustered regularly interspaced short palindromic repeat (CRISPR)‐CRISPR‐associated 9 (Cas9) nucleases were first used for genome editing in 2012, genome editing technologies have rapidly advanced. Base editors, derived from the CRISPR‐Cas system, were developed to introduce specific point mutations without requiring DNA double‐strand breaks, and subsequently, prime editing (PE) technology was created to enable insertions, deletions, and all types of point mutations. The precision and versatility of PE make it a promising tool for clinical applications. However, PE has potential limitations, including low editing efficiency and limited capacity for large‐scale manipulation. To overcome these limitations, research has been continuously conducted to improve PE efficiency and expand its capabilities. Therefore, this review aims to highlight current efforts and future directions for developing and improving PE‐related tools.

## Graphical abstract

Most rare diseases are caused by genetic mutations. Prime editing (PE) has emerged as a versatile tool capable of inducing diverse mutations without generating DNA double‐strand breaks. Despite its significant clinical potential, PE faces limitations in terms of efficiency and scalability. This review examines recent advances and future research directions aimed at improving and expanding PE‐related tools for therapeutic applications.

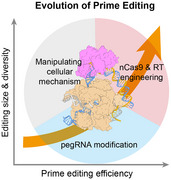

## 1 Introduction

**FIGURE 1:**
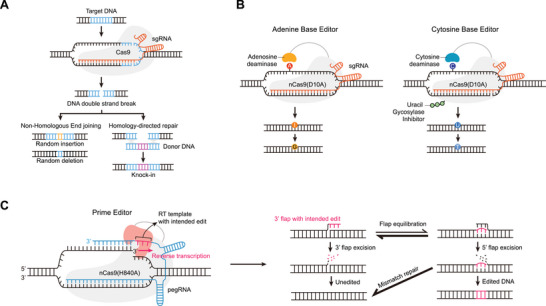
Principles of CRISPR–Cas9, base editing, and prime editing. (A) Schematic representation of CRISPR–Cas9‐mediated genome editing. DSBs generated by Cas9 are repaired through NHEJ or HDR. NHEJ directly ligates the DNA ends, often leading to random insertions or deletions, while HDR utilizes a donor DNA template containing homology arms to achieve precise knock‐in events. (B) BE architectures and mechanisms. CBE comprising a nCas9(D10A) fused to a cytidine deaminase and a UGI, mediating C•G‐to‐T•A base conversions. ABE contains an adenosine deaminase fused to nCas9 to catalyze A•T‐to‐G•C conversions. (C) PE architecture and mechanisms. The nCas9(H840A)–RT fusion is guided by a pegRNA that contains a PBS and RTT. After nicking the non‐target strand, the PBS anneals to the 3′ end of the cleaved DNA, enabling RT‐mediated synthesis of a 3′ flap containing the intended edit. DNA repair or replication, followed by flap equilibration, 5′ flap excision, and ligation, results in the edited or unedited DNA sequence. Abbreviations: ABE, adenine base editor; CBE, cytosine base editor; DSB, double‐strand break; HDR, homology‐directed repair; NHEJ, non‐homologous end joining; nCas9(D10A), Cas9 nickase with D10A mutation; nCas9(H840A), Cas9 nickase with H840A mutation; PBS, primer binding site; PE, prime editing; pegRNA, prime editing guide RNA; RT, reverse transcriptase; RTT, reverse transcription template; UGI, uracil glycosylase inhibitor.

The clustered regularly interspaced short palindromic repeat (CRISPR)‐CRISPR‐associated (Cas) system is an adaptive immune mechanism in bacteria and archaea [1, 2]. After acquiring viral DNA fragments, CRISPR effectors—such as Cas9 protein with CRISPR RNAs (crRNAs) and trans‐activating crRNA (tracrRNA)—recognize and cleave matching viral sequences in subsequent infections. This mechanism was later adapted into a powerful, programmable gene‐editing tool for higher organisms [3, 4, 5]. For effective gene editing, a single‐guide RNA (sgRNA) was created by fusing crRNA and tracrRNA. In eukaryotic cells, Cas9/sgRNA‐induced DNA double‐strand breaks (DSBs) engage two major repair pathways: nonhomologous end joining (NHEJ) and homology‐directed repair (HDR) (Figure 1A) [6]. NHEJ directly ligates broken DNA ends but often introduces random insertions and deletions (indels), causing disruption of gene function and knockout. In contrast, HDR repairs DSB sites with high fidelity by copying the cognate sequence of the donor template, enabling gene correction and knock‐in. Consequently, Cas9‐mediated genome editing can destroy or repair pathogenic genes via different repair pathways, offering therapeutic potential for genetic diseases.

However, Cas9‐mediated DSB‐based genome editing has shown potential limitations. For example, Cas9‐mediated DSB can cause large DNA deletions (> 100 base‐pairs) via an alternative end‐joining pathway, such as DNA polymerase theta‐mediated end joining (TMEJ), leading to extensive genomic loss [7, 8, 9, 10]. These DSBs can also trigger chromosomal‐scale structural variations beyond local mutations. Aberrant linkages between Cas9 cleavage sites or fusions with spontaneous DSB sites can lead to complex genomic rearrangements, including chromothripsis [9, 11, 12, 13]. Furthermore, de novo telomere formation at cleavage sites has been reported to cause widespread chromosome loss [14]. Additionally, Cas9‐induced DNA cleavage activates p53, triggering programmed cell death (or apoptosis) in mammalian cells to prevent damaged cells from dividing and potentially becoming cancerous [15, 16]. Furthermore, repetitive Cas9 cleavage can induce cellular aging and senescence, especially in blood stem cells [17]. These effects result from on‐target DNA cleavage, not off‐target activity. In this context, the development of precise genome editing tools has been moving toward approaches that do not require DSBs.

As an alternative, base editors (BEs) were developed to enable precise base conversions without creating DSBs and are classified into two main types: cytosine base editor (CBE) and adenine base editor (ABE) [18, 19]. In CBE, catalytically dead Cas9 (dCas9) or Cas9 nickase (nCas9) carrying the D10A mutation in the RuvC domain, i.e., nCas9(D10A), is fused to a cytidine deaminase, which converts cytosine (C) to uracil (U), and to an uracil DNA glycosylase inhibitor (UGI) to enhance base editing efficiency (Figure 1B). Similarly, ABE comprises an adenosine deaminase that converts adenine (A) to inosine (I) linked to dCas9 or nCas9 (Figure 1B). Ultimately, CBEs convert G·C to A·T, and ABEs convert A·T to G·C. Approximately 60% of pathogenic mutations are point mutations [20], and CBEs and ABEs could theoretically correct up to 36% of these. However, BEs also have limitations, including bystander editing—unintended modification of DNA bases near the intended target base within the editing window [21]—and various off‐target editing, such as genome‐wide and transcriptome‐wide off‐target deamination [22].

Therefore, the persistent challenges of DSB‐based editing and the limitations of BEs necessitate the development of a next‐generation technology that enables precise, high‐fidelity genome editing and, furthermore, the handling of large genomic information. Prime editing (PE) technology has been developed to address this issue. This review comprehensively examines the evolution of PE technology, covering safety considerations and recent research trends in clinical applications. We also explore novel editing modalities using a dual‐PE system, which have significantly expanded the scope of precision genome engineering.

## 2 Advent of Prime Editors and Strategies to Enhance Prime Editing Efficiency

In 2019, Liu and co‐workers first reported PE, in which nCas9 with an H840A mutation in the HNH domain is fused to a reverse transcriptase (RT) and guided by a prime editing guide RNA (pegRNA) (Figure 1C) [23]. The pegRNA, a modified sgRNA, carries a 3′ extension comprising a primer binding site (PBS) and reverse transcriptase template (RTT). The mechanism of PE involves the following steps. The nCas9 induces a DNA single‐strand break on the protospacer adjacent motif (PAM)‐containing strand, leading to exposure of single‐stranded DNA. The 3' end of the exposed single‐stranded DNA is hybridized with the PBS of the pegRNA, initiating RT‐mediated DNA synthesis. This structure contains two overlapping single‐stranded DNA flaps: a 5′ flap that retains the unedited DNA sequence and a 3′ flap that contains the desired editing sequence copied from the RTT of the pegRNA. Equilibrium occurs between these 3′ and 5′ flaps. The 5′ flap may be a preferred substrate for structure‐specific enzymes such as flap endonuclease 1 (FEN1), which is known to excise 5′ flaps generated during lagging‐strand synthesis and long‐patch base excision repair. Furthermore, 5′ exonucleases, such as EXO1, can also remove 5′ flaps. Theoretically, ligation of the 3′ flap containing the desired edit after removal of the 5' flap would allow stable incorporation of the new DNA strand. This process ultimately creates a DNA heteroduplex consisting of one strand with the desired edit and the non‐edited (i.e., original) complementary strand. Repair of this DNA heteroduplex results in either edited or unedited DNA [23]. PE‐mediated mutations are determined by the RTT sequence, which can encode all mutation types, including insertions, deletions, and point mutations. Accordingly, the high flexibility of PE enables correction of approximately 89% of pathogenic mutations listed in the ClinVar database in principle [23]. Owing to these advantages, PE has gained significant attention in academia and industry; however, its relatively low efficiency remains a major limitation, driving ongoing extensive research to enhance its performance.

### 2.1 Protein Engineering

#### 2.1.1 PE2/PEmax/PEmax^**^ Versions: Improving Prime Editing Efficiency through Protein Engineering

**FIGURE 2:**
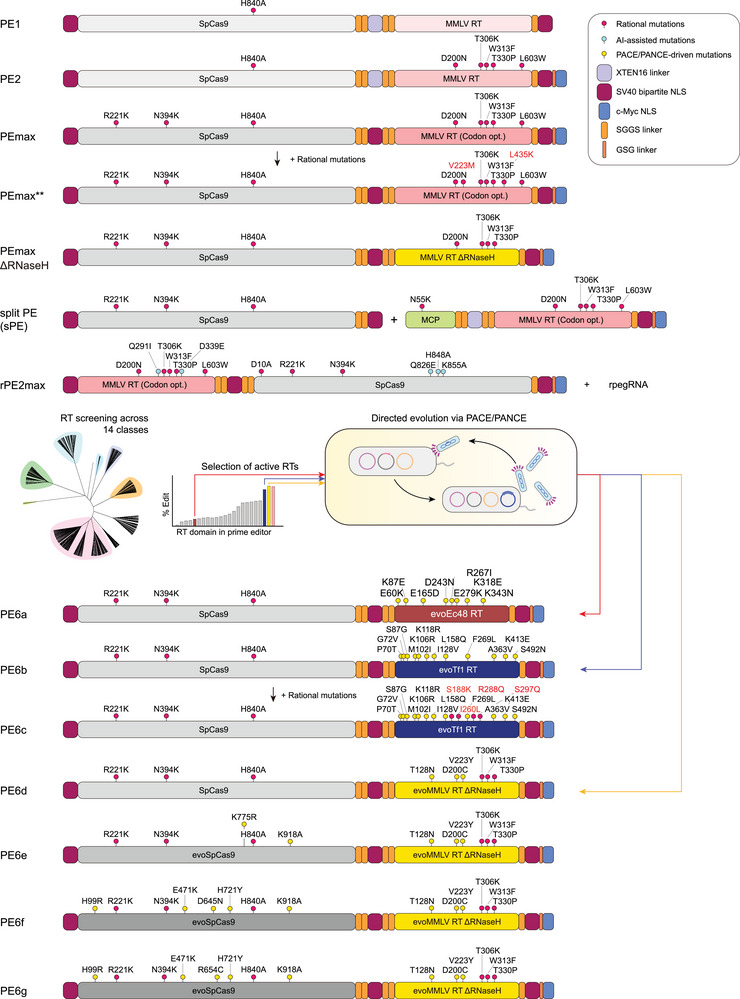
Evolution of prime editing platforms through protein engineering. Schematic representation of prime editor architectures. Introducing M‐MLV RT mutations that enhance thermostability, processivity, and substrate affinity resulted in the development of PE2. PEmax was generated through nCas9 engineering, codon optimization, and the introduction of multiple NLSs. PEmax** was engineered by incorporating mutations into PEmax that enhance its affinity for dNTPs and increase overall protein stability. PE6 variants derived through PACE/PANCE of Ec48 RT, Tf1 RT, M‐MLV RT without RNase H domain, and nCas9, producing improved versions (PE6a–g). rPE was engineered by incorporating mutations into the HNH and RT domains, which were prioritized through protein language model (PLM)‐guided variant prediction. sPE is characterized by an untethered configuration, where the nCas9 and RT are expressed as separate components. Abbreviations: M‐MLV, Moloney murine leukemia virus; NLS, nuclear localization signal; nCas9, Cas9 nickase; ngRNA, nicking guide RNA; PACE, phage‐assisted continuous evolution; PANCE, phage‐assisted non‐continuous evolution; pegRNA, prime editing guide RNA; PE, prime editing; RT, reverse transcriptase.

The initial version of the prime editor, PE1, comprised nCas9 (H840A), which nicks the non‐target DNA strand, fused to an RT derived from Moloney murine leukemia virus (M‐MLV) (Figure 2) [23]. The developers of PE1 proposed that increasing M‐MLV RT activity could enhance the overall efficiency of PE. To this end, they introduced mutations into M‐MLV RT that increase its thermal stability, processivity, and DNA–RNA substrate affinity, thereby creating PE2 (Figure 2). Owing to the enhanced activity of the engineered M‐MLV RT, PE2 showed a substantial increase in single‐nucleotide editing efficiency compared with PE1. Furthermore, by introducing mutations that enhance nCas9 function, adding extra nuclear localization signals (NLSs), and optimizing codons, PEmax, a significantly improved version, was developed (Figure 2) [24]. To further enhance the prime editor activity, Wolfe and co‐workers pursued additional protein engineering of the M‐MLV RT. Hypothesizing that intracellular dNTP concentrations might be a limiting factor for RT processivity, they introduced a V223M mutation into the active site of RT. This substitution has been reported to decrease the K_m_ value (or Michaelis constant) for dNTPs by two‐ to fourfold, thereby improving catalytic efficiency under physiological conditions. Subsequently, a L435K mutation was incorporated to increase the solubility of the RT domain. By integrating these two mutations into the PEmax architecture, they developed PEmax^**^, which demonstrated significant improvement in PE efficiency across various target sites compared to the original PEmax (Figure 2) [25].

#### 2.1.2 PE6 Versions: Improving Prime Editing Efficiency Through Protein Evolution

For PE architecture, RT enzyme activity is critical for PE activity; therefore, developing new RT variants could enhance PE efficiency. In the initial version, PE2, previously identified beneficial mutations were introduced into M‐MLV RT. However, Liu and co‐workers further engineered nCas9 and ortholog RTs using phage‐assisted continuous evolution (PACE) and phage‐assisted non‐continuous evolution (PANCE), which employ bacteriophages to rapidly modify target genes under defined selection pressures [26]. Ultimately, they generated PE6 series [27] by engineering two RTs smaller than M‐MLV RT— *Escherichia coli* Ec48 retron RT (Ec48 RT) and *Schizosaccharomyces pombe* Tf1 retrotransposon RT (Tf1 RT)—via PANCE, producing enhanced variants evoEc48 RT and evoTf1 RT. PE6a incorporated evoEc48 RT (Figure 2), and PE6b incorporated evoTf1 RT (Figure 2) [27]. Additional beneficial mutations enhancing Tf1 RT function were incorporated into PE6b, producing PE6c (Figure 2). They also applied PACE/PANCE to RNase H‐deficient M‐MLV RT (ΔRNase H), producing an improved variant used in PE6d (Figure 2) [27]. Protein evolution was also applied to nCas9. Evolving the PE2 via PACE/PANCE revealed nCas9 mutation combinations that significantly enhanced PE efficiency, producing PE6e, PE6f, and PE6g (Figure 2) [27]. The selection of the optimal PE6 variant is determined primarily by editor size constraints and the characteristics of the desired edit, particularly the length and secondary structure of the RTT. For strict size minimization, PE6a is the smallest available prime editor, while PE6b is preferred for edits with short, unstructured RTTs to reduce unwanted pegRNA scaffold incorporation. Conversely, PE6c and PE6d offer enhanced activity and processivity, making them optimal for challenging edits, including twinPE, or those using pegRNAs with highly structured RTTs. Finally, the Cas9 domain variants from PE6e–g can be modularly screened in combination with an optimized RT to further enhance editing efficiency for specific genomic targets.

#### 2.1.3 rPE: AI‐Driven Protein Optimization

To date, engineering strategies for prime editing have predominantly relied on rational design—informed by structural insights and empirical data regarding mutational impacts—or continuous directed evolution. A recent study [28] on reverse prime editing (rPE) illustrates how artificial intelligence (AI) can accelerate protein engineering to advance the PE landscape. rPE uses nCas9(D10A) and RT to generate a “reverse” editing window at the 3′ end of the HNH‐mediated nick site, complementing the canonical RuvC‐directed PE window. Systematic optimization of rpegRNA architecture, the HNH domain, and M‐MLV RT yielded rPE variants with robust editing across multiple genomic loci and edit types. To further improve rPE, Yang and coworkers applied Evolutionary Scale Modeling (ESM [29]) based protein language models (PLMs) to prioritize beneficial mutations in both HNH and M‐MLV RT. PLM‐guided variant prediction followed by targeted experimental validation identified substitutions such as Q826E in HNH and Q291I/D339E in RT that significantly enhanced PE efficiency (Figure 2). Incorporation of codon optimization enhanced nuclear localization signals, additional structural improvements, and eventually the La protein resulted in erPE7max, which exhibited relatively high PE efficiency.

#### 2.1.4 Split Prime Editing (sPE) System for Enhanced Delivery

Despite its versatility, the broad therapeutic application of PE is significantly hindered by its substantial molecular size and structural complexity. The fusion of a large nCas9 protein with RT creates a bulky molecular machinery that frequently exceeds the cargo limit (approximately 4.7 kb) of adeno‐associated virus (AAV) vectors, which are widely adopted platforms for in vivo gene delivery. As an alternative approach, Xue, Sontheimer, and coworkers reported a split PE (sPE) system where the nCas9 and RT were not tethered (Figure 2) [30]. The sPE system, based on MS2 and SunTag mechanisms, demonstrated comparable editing efficiency to the conventional tethered PE system. Furthermore, in in vivo plasmid delivery experiments via hydrodynamic tail vein injection, the sPE showed superior efficiency compared to the tethered configuration. Applying this sPE approach via dual‐AAV delivery, nCas9 was packaged into one AAV, while the pegRNA and RT were delivered by the other AAV, demonstrating therapeutic benefits in Fah‐mutant mice. This sPE system, utilizing a Cas protein and an untethered RT, was optimized by incorporating circularized RNA, as detailed in the subsequent sections [30].

### 2.2 Regulating Cellular Mechanism

#### 2.2.1 PE3 Version: Enhancing Prime Editing Efficiency by Introducing an Additional Nick in the Non‐edited DNA Strand

**FIGURE 3:**
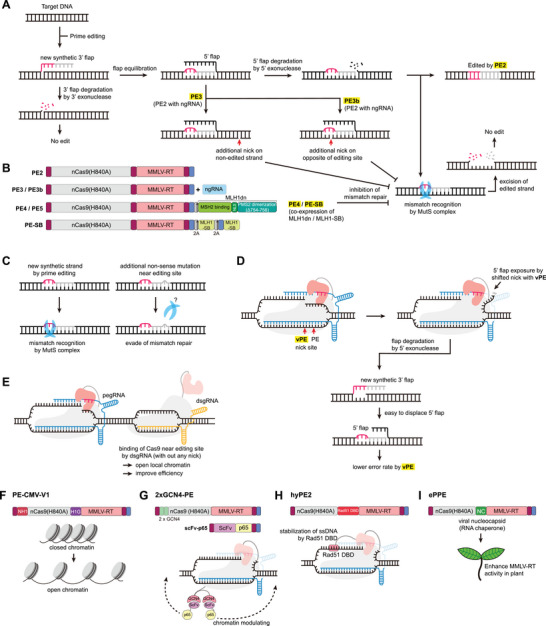
Strategies to enhance PE by regulating cellular mechanisms. (A) PE3 strategy introducing an additional nick on the non‐edited strand to promote incorporation of the edited DNA during heteroduplex resolution. Schematic representation of PE3b. PE generates a 3′ flap DNA strand carrying the intended edit (red). The ngRNA recognizes this newly synthesized strand and induces a nick on the opposite, non‐target strand after successful incorporation of the desired edit. (B) Architectures of PE2, PE3/PE3b, PE4/PE5, and PE‐SB. Co‐expressing or fusing a dominant‐negative MLH1 (MLH1dn) or MLH1‐SB suppress mismatch repair and thereby enhance editing efficiency. (C) Introduction of additional non‐sense mutations could enhance PE editing by evading MMR. (D) Schematic of the vPE mechanism, where relocating the nick position in a PAM‐distal direction suppresses unintended indel formation during prime editing. (E) dsgRNA targeting near PE target site could lead a modulation of local chromatin and improve PE efficiency. (F) Chromatin modulation by chromatin‐modulating peptides from the HN1‐CMP and H1G‐CMP. (G) Co‐expression of the prime editor with p65 increases chromatin accessibility and editing efficiency. (H) The Rad51 DBD stabilizes the single‐stranded DNA generated during nicking. (I) The viral NC protein functions as a nucleic acid chaperone, promoting annealing between the target DNA and the PBS of the pegRNA, thereby stabilizing the DNA–RNA hybrid. Abbreviations: PE, prime editing; RT, reverse transcriptase; M‐MLV, Moloney murine leukemia virus; NLS, nuclear localization signal; nCas9, Cas9 nickase; ngRNA, nicking guide RNA; pegRNA, prime editing guide RNA; MLH1dn, dominant‐negative MLH1; MLH1‐SB, MLH1 small binder; PE‐SB, prime editor fused with MLH1‐SB; dsgRNA, dead sgRNA; HN1‐CMP, high‐mobility group nucleosome‐binding domain chromatin‐modulating peptide; H1G‐CMP, H1 central globular domain chromatin‐modulating peptide; DBD, DNA‐binding domain; NC, nucleocapsid.

In the PE mechanism (Figure 3A), the newly synthesized DNA forms a 3′ flap that displaces the original strand to expose a 5′ flap for genomic incorporation. The exposed 5′ flap is prone to excision, resulting in a heteroduplex of the edited strand and non‐edited complementary strand. This heteroduplex contains mismatched bases; consequently, repair may result in either successful mutation incorporation or restoration of the original sequence, depending on which DNA strand is removed. To enhance PE efficiency by promoting non‐edited strand removal, PE3 adds a nicking guide RNA (ngRNA) to the PE2 system, inducing an additional single‐strand break in the target strand (Figure 3A,B). PE3 showed increased efficiency over PE2. However, because PE3 introduces a single‐strand break in both the non‐target (or edited) strand and target strand, it substantially increases undesired indel mutations compared with PE2. In PE3b, ngRNAs are designed such that the spacer matches the desired edit sequence but does not match the original sequence (5' flap) on the edited strand, typically by creating a new PAM or altering the seed position within the second protospacer of the edited strand (Figure 3A,B). This configuration enriches nicking events that occur after the edited strand is incorporated, thereby reducing the chance of simultaneous nicks on both strands. Consistent with this design, PE3b showed similar PE efficiencies but an approximately 13‐fold lower indel frequency than PE3 in the original study [23].

#### 2.2.2 PE4/PE5 Versions: Improving Prime Editing Efficiency by Inhibiting Mismatch Repair

Genome editing, including PE, is closely linked to DNA repair processes [31, 32]. To investigate how cellular DNA repair pathways influence PE, Liu and coworkers performed a CRISPR interference (CRISPRi)‐based screen targeting 476 DNA repair‐related genes to identify PE‐associated repair pathways, including mismatch repair (MMR) [24]. The screen showed that inhibiting MMR‐associated genes— MSH2, MSH6, MLH1, and PMS2—increased PE efficiency. Based on this, a set of 55 dominant‐negative proteins was co‐expressed with PE2 to identify a suitable MMR inhibitor. Among them, the dominant‐negative human MLH1 variant lacking amino acids 754–756 (MLH1dn) significantly increased PE efficiency, showing the best performance. MLH1dn was subsequently fused to the N‐terminus of PE2 via a 2A self‐cleaving peptide to create PE4, which substantially enhanced efficiency (Figure 3B). Similarly, fusing MLH1dn to PE3 with a 2A self‐cleaving peptide produced PE5, which moderately increased the efficiency of PE3 (Figure 3B) [24]. Notably, independent study had also validated that suppressing MMR robustly improves PE outcomes, aligning with the mechanistic rationale underlying PE4 and PE5 design [33, 34].

#### 2.2.3 PE‐SB Platform: Improving Prime Editing Efficiency through Artificial Intelligence‐Generated Small Binders

As shown during PE4/5 development, cellular MMR inhibits PE [24], promoting the widespread use of MLH1dn to enhance PE efficiency. However, MLH1dn, comprising 752 amino acids, and its large size pose challenges for in vivo delivery. Bae and co‐workers recently harnessed advances in AI to develop a novel strategy for MMR inhibition (Figure 3B) [35]. MLH1 small binders (MLH1‐SBs) were generated using RFdiffusion, a generative AI‐based de novo protein design tool [36]. These binders target the C‐terminal domain of MLH1, its dimerization interface with PMS2. Additionally, AlphaFold 3 was employed to design a “competition test” to filter MLH1‐SBs predicted to have low binding activity [35]. The selected MLH1‐SBs, only 82 amino acids, successfully increased PE efficiency by disrupting the MLH1‐PMS2 complex, outperforming MLH1dn. Additionally, MLH1‐SB is compatible with preexisting PE systems, including PE2, PE3, PE6, and PE7. The PE‐SB platform was created by combining MLH1‐SB with these editors via 2A peptides (Figure 3B). PE7‐SB2, with two MLH1‐SBs linked to PE7 via 2A peptides, showed a significant increase in efficiency over PEmax and PE7 in human cells.

#### 2.2.4 Enhancing Prime Editing Efficiency through Synonymous Mutations

In their study establishing the PE4 and PE5 systems, Liu and co‐workers introduced a strategic design for pegRNAs to enhance PE efficiency [24]. They demonstrated that the MMR‐associated machinery is significantly less effective at recognizing heteroduplexes that contain three or more consecutive mismatched nucleotides. By intentionally incorporating synonymous mutations near the target site to enlarge the ‘mismatch bubble’, they developed an MMR‐evasive editing strategy (Figure 3C) [24]. This approach markedly improves editing outcomes without the need for direct exogenous inhibition of cellular MMR pathways. Given that it relies solely on pegRNA optimization, this strategy holds substantial clinical promise for therapeutic applications where minimal intervention in global DNA repair mechanisms is preferred.

#### 2.2.5 vPE: Engineered Prime Editors with Low Indel Errors

During the PE process, the 3' end of the DNA substrate nicked by the nCas9 is released from the Cas9 complex to anneal with the PBS of the pegRNA, while the 5' end competes with the RT‐synthesized edited DNA strand [23]. However, the perfectly matched 5' flap is thermodynamically favored for annealing to the complementary strand over the edited new strand. This structural bias not only limits editing efficiency but also induces errors [24]. Recently, Langer and coworkers reported a strategy to induce the degradation of the competing 5' flap by introducing mutations into the nCas9 to facilitate nick position relaxation (Figure 3D) [37]. Shifting the nick position in the PAM‐distal direction significantly increased the degradation of the 5' flap, thereby drastically reducing the rate of indel mutations generated during PE. The resulting PE, termed vPE, exhibited editing efficiencies comparable to the conventional PE systems but with a significant reduction in indel errors.

#### 2.2.6 Enhancing Prime Editing Efficiency via Regulating Accessibility and Processability

**FIGURE 4:**
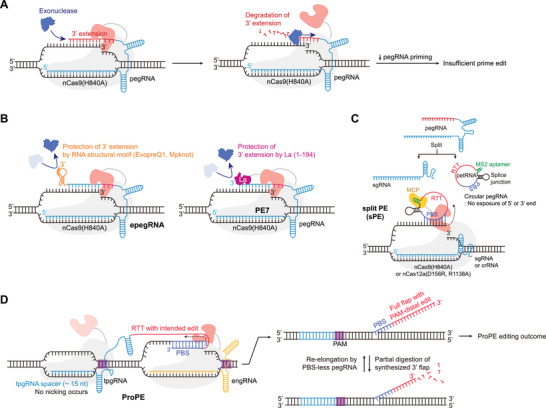
Enhancement of PE efficiency via pegRNA protection and structural modulation (A) Susceptibility of the pegRNA 3' extension to intracellular exonucleases as a primary bottleneck for prime editing efficiency. (B) Modified pegRNAs containing structured RNA motifs—including *mpknot*, *evopreQ1* (epegRNA)—designed to protect the 3′ extension from exonuclease‐mediated degradation. RNA motifs are shown in orange (left). (B) Schematic representation of La protein‐mediated PE enhancement. The La protein prevents exonuclease‐mediated degradation of the pegRNA 3′ ends and forms the PE7 system when combined with PEmax (right). (C) Structural architecture of the circRNA‐based sPE system. In this system, the RTT‐PBS is uncoupled from the sgRNA and circularized to form a circular RNA molecule. This circularization effectively shields the RTT‐PBS from exonuclease‐mediated degradation. (D) ProPE separates RT template delivery from DNA nicking by using a non‐cleaving pegRNA, tpgRNA, and a standard sgRNA, engRNA, respectively. This configuration bypasses the inhibitory effects of PBS‐spacer binding and protects the PBS from degradation, thereby sustaining high prime editing performance. Abbreviations: nCas9, Cas9 nickase; pegRNA, prime editing guide RNA; mpknot, frameshifting pseudoknot from M‐MLV; evopreQ1, modified prequeuosine1‐1 riboswitch aptamer; epegRNA, engineered pegRNA; RT, reverse transcriptase; La, La protein; sPE, split Prime Editing system; nCas12a, Cas12a nickase; ProPE, prime editing with prolonged editing window; tpgRNA, template providing guide RNA; engRNA, essential nicking guide RNA.

Efficient gene editing requires effective access of the prime editor/pegRNA complex to the target DNA site. Chromatin accessibility of the prime editor depends on the surrounding chromatin environment. To enhance PE efficiency, Kim and co‐workers developed a strategy to modulate the chromatin landscape neighboring the target site [38]. Their approach involved the co‐administration of 14‐ or 15‐nt dead sgRNAs (dsgRNAs) designed to bind near the target locus, which facilitates local chromatin remodeling (Figure 4E). Furthermore, they engineered prime editor by fusing it with chromatin‐modulating peptides (CMPs) derived from the high‐mobility group nucleosome‐binding domain 1 (HN1) and the histone H1 central globular domain (H1G) (Figure 4F). These dual modifications—both RNA‐guided and protein‐tethered—synergistically increased chromatin accessibility, leading to a significant improvement in PE performance across various genomic contexts (Figure 4E,F) [38]. Similarly, treatment with a histone deacetylase 7 (HDAC7) inhibitor increases PE efficiency by inducing chromatin opening [39]. Additionally, the transcriptional activator p65 binds nucleosomes and facilitates chromatin decondensation [40]. Zhang and coworkers report that co‐expressing the prime editor with p65 increases chromatin accessibility and, consequently, editing efficiency (Figure 4G) [41]. Using a PE reporter system, Shendure and co‐workers show the effect of the chromatin context on PE efficiency [42]. They report that PE efficiency positively correlates with the H3K79me2 marker but negatively with the H3K9me3 marker. They also report that active transcription elongation increases PE efficiency, which can be altered using CRISPR‐mediated gene silencing or activation. Together, these findings suggest that the chromatin environment influences PE efficiency, and it can be enhanced through chromatin modulation.

Incorporating auxiliary proteins can further enhance PE efficiency by stabilizing intermediate steps in the editing process. Kim and coworkers designed hyPE2, which integrates the Rad51 DNA‐binding domain into the linker region to stabilize the single‐stranded DNA generated by nCas9 (Figure 4H) [43]. hyPE2 exhibit enhanced efficiency compared to that of PE2, showing 1.4‐ and 1.5‐fold increases in HEK293T and HCT116 cell lines, respectively. Gao and coworkers developed the engineered plant prime editor (ePPE), which enhances PE efficiency in plants by deleting the RNase H domain from M‐MLV RT and inserting a viral nucleocapsid (NC) protein into the linker region (Figure 4I) [44]. In rice protoplasts, ePPE shows an average of 5.8‐fold increase in PE efficiency. The NC protein likely contributes to this improvement through its nucleic acid chaperone activity, which facilitates reverse transcription.

### 2.3 pegRNA Protection and Modulation

#### 2.3.1 Protection of pegRNA 3’ End by Structured RNA Motifs

As discussed above, continuous engineering of Cas proteins and RT components aims to improve PE efficiency. Beyond protein modifications, substantial research also focuses on improving the pegRNA. Liu and coworkers report that the degradation of the 3' extension of pegRNA, which is not protected by the nCas9, is a limiting factor for PE efficiency (Figure 4A) [45]. To protect the 3' extension from intracellular exonucleases, they improve pegRNA stability by attaching structured RNA motifs to its 3′ terminus. The engineered pegRNAs (epegRNAs) incorporate either a modified prequeuosine1‐1 riboswitch aptamer (evopreQ1) or the frameshifting pseudoknot from M‐MLV (mpknot) at the 3′ end (Figure 4B) [45]. However, the linker sequences connecting these motifs often disrupts pegRNA function. To address this, the authors developed the pegRNA Linker Identification Tool, which computationally designs optimal linkers to ensure consistent improvement in epegRNA performance. Consequently, protecting the 3′ end of pegRNA with these structures increases PE efficiency across multiple targets compared to those of conventional pegRNAs. Similarly, several independent research groups enhance PE efficiency by attaching diverse structural RNA motifs to the 3' extension of the pegRNA. These include the Zika virus exoribonuclease‐resistant RNA motif (xrRNA) [46], the G‐quadruplex (G‐PE) [47], and the stem‐loop aptamer (sPE) [48]. Collectively, these findings underscore the vital role of intracellular pegRNA stability in determining PE efficiency.

#### 2.3.2 PE7 Version: Improving Prime Editing Efficiency through 3′‐End Protection of pegRNA

Adamson and co‐workers expanded CRISPRi screening from 476 DNA repair‐related genes to 18905 genome‐wide genes to identify additional factors influencing PE efficiency [49]. This screen showed that La protein deficiency significantly reduced PE efficiency, indicating the role of La in enhancing it (Figure 4B). The 3′ extension of the pegRNA, unlike sgRNA, remains exposed when bound to the prime editor protein, making it susceptible to exonuclease degradation [45]. The La protein, a small RNA‐binding exonuclease protection factor widely expressed in eukaryotic cells, binds the poly‐U tract at the 3′ end of RNA polymerase III‐transcribed pegRNAs, protecting them from exonuclease‐mediated degradation [49, 50]. Based on this observation, fusing La protein to the C‐terminus of PEmax created PE7, which showed significantly higher efficiency than PEmax (Figure 4B). Furthermore, beyond its primary function of 3' end protection, La protein may influence PE through other mechanisms, such as nuclear retention of pegRNAs, effector complex formation, or target‐dependent degradation mediated by RNase H [49].

#### 2.3.3 Circular pegRNA

Xue, Sontheimer, and coworkers attempted to separate the pegRNA into an sgRNA component and an RTT‐PBS component in their sPE system described above (Figure 2) [30]. The RTT‐PBS component was engineered to associate with untethered RT through the incorporation of an MS2 aptamer, and was subsequently circularized to enhance resistance to RNases and improve RNA stability (Figures 2 and 4C). This circularized form was named prime editing template RNA (petRNA). petRNA demonstrated a substantially higher editing efficiency compared to its linear counterpart and exhibited efficiency comparable to conventional pegRNA. When comparing the intracellular RNA abundance, the circularized petRNA showed a much greater abundance and integrity than the linear form of pegRNA. Gao and coworkers adapted the PE system to Cas12a using circular RNA (Figure 4C) [51]. They engineered LbCas12a originating from the *Lachnospiraceae bacterium* by introducing a R1338A mutation to create a nickase and a H759A mutation to eliminate intrinsic RNA cleavage activity, ultimately yielding rnCas12a. They configured the circular RNA‐mediated prime editor (CPE) system by concurrently utilizing a crRNA for target specification and a circular RNA containing the RTT‐PBS sequence. Moving forward, they also developed a split nickase‐dependent CPE (sniCPE) system, which employs an untethered RT. Circular RNA forms not only possess resistance to RNA exonuclease but also the ability to bind to specific genomic regions to form an R‐loop [52, 53]. Building upon this advantage of circular RNA, Gao and coworkers further devised the circular RNA‐mediated inverse prime editor (ciPE), which utilizes nCas9 (D10A), an untethered RT, and a circular RTT‐PBS (Figure 4C) [54]. The ciPE system exhibited significantly enhanced PE efficiency compared to its earlier version, the inverse prime editor (iPE).

#### 2.3.4 Prime Editing with Prolonged Editing Window (ProPE)

Welker and coworkers sought to overcome phenomena that inhibit the efficiency of conventional PE by employing two separate guide RNAs (gRNAs) (Figure 4D) [55]. The essential nicking guide RNA (engRNA) functions as a conventional gRNA, which the prime editor uses to introduce a nick at the target site. The template‐providing guide RNA (tpgRNA) is similar in structure to the conventional pegRNA, possessing both RTT‐PBS sequence. However, the tpgRNA binds adjacent to the target site and serves to present the RTT‐PBS sequence near the nicked DNA strands. Crucially, the tpgRNA's spacer is truncated (11–15 nucleotides), ensuring that the nCas9 remains in an inactive form and only performs the function of binding to the DNA. The proposed PE with prolonged editing window (proPE) system mitigates efficiency losses associated with PBS instability and self‐complementarity between the PBS and spacer sequences; consequently, it generally improved overall PE efficiency, specifically for low‐performing edits. Furthermore, proPE exhibited an expanded modification range compared to typical prime editors, thereby significantly enhancing its applicability for pathogenic mutations.

## 3 Large‐Scale DNA Manipulation using Prime Editing

A key limitation of PE technology is its restricted edit size. PE systems efficiently generate base substitutions and small insertions or deletions, typically within approximately 40 base‐pairs (bp). This flexibility enables the correction of approximately 89% of known human pathogenic variants listed in ClinVar, underscoring the high potential of PE for gene therapy. However, disease‐causing genetic variants vary widely, ranging from single‐base substitutions to large‐scale deletions, duplications, and rearrangements spanning megabases [20]. Therefore, developing approaches that can correct a broad spectrum of large‐scale variants and expand the capacity of PE to accommodate larger DNA edits, including gene‐length sequences, is essential to address these limitations.

Previously, large‐scale genomic deletions were generated using paired sgRNAs with wild‐type Cas9. This approach leverages the NHEJ pathway to induce targeted deletions or inversions [56]. However, this method often results in imprecise editing due to indel formation. When donors are available, other strategies, such as homology‐independent targeted integration (HITI) and HDR, have also been explored for inserting large genetic sequences, but both remain limited. Both HITI and HDR still induce indel formation, and HDR is inherently inefficient and cell cycle dependent, making it primarily applicable only to actively dividing cells [57]. Recently, PE has emerged as a promising solution to these challenges, enabling the precise manipulation of large‐scale genetic information with high fidelity.

### 3.1 Utilization of Paired pegRNAs

**FIGURE 5:**
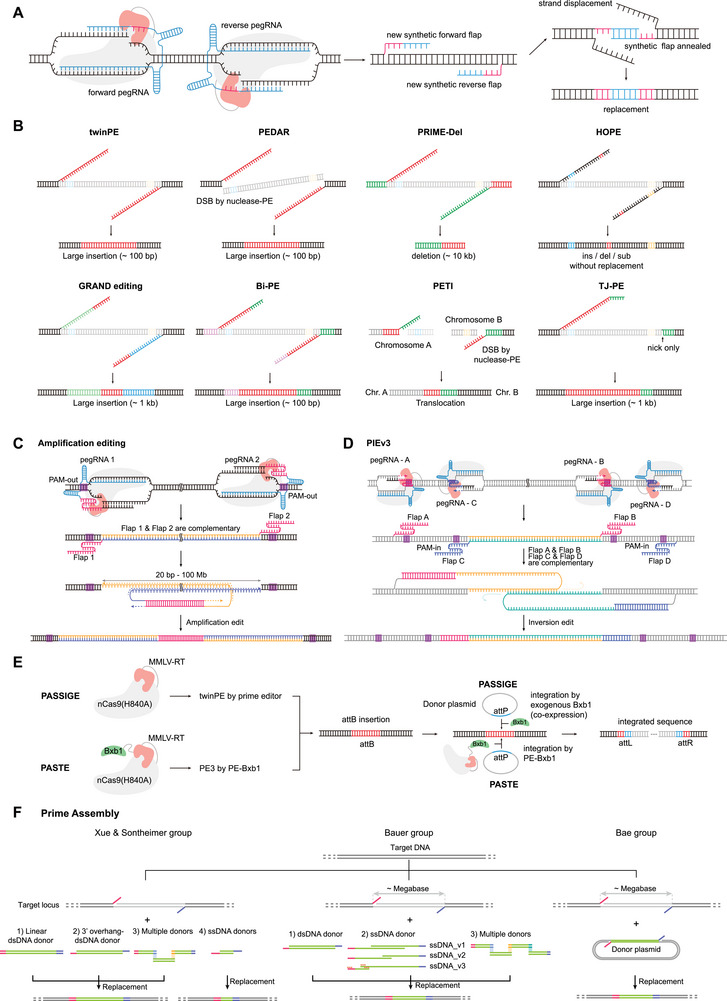
Expanding prime editing capacity for large‐scale genomic modifications. (A) Mechanism of dual‐pegRNA‐mediated prime editing. Two pegRNAs are designed to target opposite DNA strands, generating two newly synthesized 3' flaps via reverse transcription. These flaps undergo inter‐strand hybridization to function as a secondary template, facilitating high‐efficiency insertion and deletion outcomes. (B) PE utilizing dual pegRNAs employs a PAM‐in orientation to target opposite DNA strands, generating complementary extensions. These extensions facilitate the formation of hybridized flaps, which serve as a template for mediating precise genomic deletions or large‐scale insertions.TJ‐PE employs a secondary primer‐binding site on the pegRNA to allow template switching during reverse transcription, facilitating the insertion of sequences. (C) AE synthesizes complementary 3′ flaps in the reverse direction compared to the twinPE. Annealing of these flaps, followed by DNA synthesis and ligation, results in amplification of DNA sequences between the 3′ flaps, up to 100 Mbp. (D) PIE utilizes two pairs of pegRNAs designed to generate 3' flaps that are complementary to each other, thereby mediating the inversion of the intervening DNA sequence through flap hybridization. (E) PASSIGE and PASTE install recombinase recognition sites (attB/attP) via twinPE or standard PE. Bxb1‐mediated integration of donor DNA enables targeted gene insertions. (F) PA enables large and precise DNA insertions through complementary flaps (Flap A′ for Flap A and Flap B′ for Flap B) on both genome and donor templates, allowing efficient assembly with plasmid or linear donors. Abbreviations: PE, prime editing; pegRNA, prime editing guide RNA; twinPE, twin prime editing; PEDAR, PE‐Cas9‐based deletion and repair; HOPE, homologous 3' extension‐mediated prime editor; GRAND, genome editing by partially aligned but non‐homologous RTTs within dual pegRNAs; Bi‐PE, bi‐direction prime editing; PETI, prime editor nuclease‐mediated translocation and inversion; TJ‐PE, template‐jumping prime editing; AE, amplification editing; Mbp, megabase pairs; PIE, prime‐editing‐based inversion with enhanced performance; PASSIGE, programmable addition via site‐specific integration with genome editing; PASTE, programmable addition via site‐specific targeting elements; attB/attP, bacteriophage recombination sites; PA, prime assembly.

Several groups report that paired pegRNAs can be used for PE (Figure 5A) [58, 59, 60, 61, 62, 63, 64, 65]. These paired pegRNAs are designed in a PAM‐in orientation to target opposite DNA strands, with complementary 3' extensions that allow the newly synthesized DNA flaps from each target site to anneal. The resulting DNA flaps effectively hybridize and act as secondary templates, achieving highly efficient and precise insertions and deletions. Paired pegRNA systems show greater accuracy and efficiency than those of traditional PE. For example, studies developed paired‐pegRNAs‐based approaches—such as twin prime editing (twinPE), PE‐Cas9‐based deletion and repair (PEDAR), PRIME‐Del, homologous 3' extension‐mediated prime editor (HOPE), genome editing by partially aligned but non‐homologous RTTs within dual pegRNAs (GRAND), bi‐direction prime editing (Bi‐PE), and prime editor nuclease‐mediated translocation and inversion (PETI)—to achieve efficient, precise, and large‐scale sequence modifications (Figure 5B) [58, 59, 60, 61, 62, 63, 64, 65]. While these systems enable accurate large‐scale deletions, insertion size remains limited due to the inherent constraints of PE. Furthermore, in the case of PEDAR, which utilizes dual Cas9 nucleases, there is a high possibility of adverse effects resulting from DSBs, as previously pointed out.

### 3.2 Template‐Jumping Prime Editing

Drawing inspiration from the insertion mechanism of non‐LTR retrotransposons, Xue and coworkers developed template‐jumping prime editing (TJ‐PE), a method that utilizes a pegRNA containing a secondary PBS and ngRNA (Figure 5B) [66]. In this study, the authors demonstrate that TJ‐PE enables the insertion of a 0.8‐kb GFP cassette through PE and the generation of precise large deletions. While TJ‐PE operates via a mechanism analogous to the paired‐pegRNA system, a key distinction lies in the annealing process: whereas the paired‐pegRNA system relies on the annealing of two complementary DNA flaps both synthesized by RT, TJ‐PE functions through the annealing of a single RT‐generated flap with a genomic DNA flap produced by the nickase [58, 60, 63, 66].

### 3.3 Chromosomal Scale Duplication and Inversion using Prime Editing

Yin and coworkers developed a genome‐editing tool named Amplification Editing (AE) enables precise duplication of megabase‐scale chromosomal DNA in human cells (Figure 5C) [67]. AE employs a pair of pegRNAs arranged in a PAM‐out orientation to generate complementary 3' flaps. These flaps, synthesized by the prime editor, anneal and initiate DNA synthesis using the sequence between the two nicks as a template. Consequently, the intervening sequence is accurately duplicated, leaving a residual flap sequence between the repeats. AE achieves up to a 1 Mb duplication with 73.0% efficiency and a maximum of 100 Mb duplication with 3.4% efficiency, thereby demonstrating the feasibility of precise chromosomal‐scale genome engineering.

The same group also developed a method termed prime‐editing‐based inversion with enhanced performance (PIE) to achieve precise chromosomal‐scale inversions using prime editors (Figure 5D) [68]. They designed a pair of PAM‐in orientation pegRNAs on the outer side and a pair of PAM‐out orientation pegRNAs on the inner side at the junctions of the inversion region. Each pegRNA was designed to generate a complementary 3′ flap on the opposite DNA strand. The optimized PIEv3b version achieved inversion efficiencies of up to 61.7% for a 1 Mb fragment and 14.2% for a 50 Mb fragment. They showed that the system can invert chromosomal segments as large as 100 Mb, underscoring its potential as a robust tool for engineering chromosomal structural variations.

### 3.4 Harnessing Recombinase with Prime Editing

Paired‐pegRNA and TJ‐PE methods enable precise large‐scale deletions but are limited in insertion size due to constraints of the PE system. The stability of the 3' extension of the pegRNA decreases beyond a certain length, and structural limitations within the PE system restrict the synthesis of long DNA sequences. To address this challenge, a hybrid system combining PE with recombinases was developed to enable whole‐gene replacement. The Liu group and the Gootenberg group independently developed methods for gene‐sized insertions using a prime editor and a recombinase (Figure 5E) [59, 69]. These methods involve using a prime editor to insert a recombinase landing site (attB or attP) into the genome. A DNA donor carrying the complementary recombinase attachment site (attP or attB) is then co‐delivered with the recombinase (BxbI) to mediate site‐specific integration at the target locus (Figure 5E). The two groups named their respective methods programmable addition via site‐specific targeting elements (PASTE) and prime‐editing‐assisted site‐specific integrase gene editing (PASSIGE), demonstrating DNA insertions up to 36 kb and gene inversions up to 40 kb [59, 69]. Despite these major advances, some limitations remain. Several components are required for prime editors and recombinases, posing challenges for in vivo delivery. Moreover, insertion efficiency depends on both enzymes and varies significantly with the genomic surroundings of the landing site. This approach also leaves a junctional scar, such as an attP–attB junction, at the insertion site, which may cause unpredictable functional effects. Therefore, additional technological development is needed to achieve efficient large‐scale DNA editing suitable for clinical applications.

### 3.5 Prime Assembly

Recently, three research groups independently reported a prime assembly (PA) method that enables precise large‐scale DNA replacement (Figure 5F) [70, 71, 72]. In PA, PE was used to generate 3' flaps on the target genome and/or donor DNA. The target flap is designed to be complementary to the donor flap, allowing the two to anneal. Xue and coworkers utilized linearized double‐stranded DNA (dsDNA) donors to achieve highly accurate insertions of up to 11 kb (Figure 5F, left) [72]. They also demonstrate that insertion can occur by joining two separate donors in a manner analogous to Gibson assembly. Bauer and coworkers employed 3'‐overhang dsDNA donors with a partial double‐stranded region to reduce the cytotoxicity associated with dsDNA donors (Figure 5F, middle) [71]. They show targeted integration using PA in hematopoietic stem and progenitor cells (HSPCs). Bae and coworkers adopted a slightly different approach from the other groups (Figure 5F, right) [70]. Compared to the others, they used PE to generate genomic flaps and simultaneously created complementary flaps on the donor DNA to facilitate PA. This strategy enables efficient use of plasmid‐based donors, highlighting a key advantage in donor preparation and delivery. The PA system allows precise replacement of large‐scale genomic segments, including megabase‐scale deletions and multi‐kilobase insertions (up to 11 kb), without relying on nuclease‐driven DSBs, thereby expanding its potential for universal gene therapy applications across multiple disease contexts.

## 4 Accuracy and Safety Issues of Prime Editors

### 4.1 On‐Target Editing Fidelity

Conventional genome editing with wild‐type Cas9 induces DSBs that are frequently repaired through NHEJ, resulting in random indels. When repair fails, more severe outcomes, such as kilobase‐scale deletions, chromosomal translocations, or inversions, can occur [8, 9, 73]. In contrast, PE generally employs a nCas9 to induce a nick, substantially reducing DSB‐associated risks. A comparative study reports that PE leads to large deletions at frequencies 20‐fold lower than those observed with conventional DSB‐based editing [7]. Nevertheless, PE is not entirely free of adverse outcomes at on‐target site, as pegRNA and auxiliary ngRNAs can introduce distinct unintended edits. In the PE3 system, inclusion of an additional ngRNA increases the frequency of small indels near the edit site [23]. Variants such as PE3b, which directs the nick exclusively to the edited strand, were developed to mitigate this issue [23]. While PE largely avoids DSB‐associated errors, introducing two closely spaced nicks on opposite strands can occasionally mimic a staggered DSB. Such events may destabilize the intervening DNA and cause unintended large deletions, although at substantially lower frequencies than those observed with conventional nuclease‐based editing [7]. Similarly, paired‐pegRNA approaches such as twinPE and PRIME‐Del are intentionally designed to induce predictable large deletions, inversions, or other rearrangements [59, 60]. While these methods expand the functional scope of PE and enable programmable structural modifications, they also increase the risk of unintended structural variants compared with single‐pegRNA editing.

### 4.2 Concerns Regarding Off‐Target Effects

As PE utilizes nCas9 instead of the conventional Cas9 nuclease, its off‐target activity is significantly lower than that of conventional DSB‐based genome editing [23, 74]. Genome‐wide analyses using techniques such as nickase‐based in vitro Cas9‐digested whole‐genome sequencing (nDigenome‐seq), TAgmentation of Prime Editor sequencing (TAPE‐seq), and the genome‐wide prime editor off‐target detection system (PE‐tag) demonstrate that unintended PE events occur at low frequencies [74, 75, 76]. The off‐target potential of PE is determined using sequence complementarity to the spacer region and homology to the RTT and PBS. These additional sequence constraints further minimize off‐target editing [74]. Furthermore, incorporating a secondary ngRNA in the PE3 system does not significantly increase off‐target events, suggesting that PE exhibits higher specificity than nuclease‐based CRISPR approaches [74]. Additionally, whole‐genome analyses have not detected any increase in gRNA‐independent mutations attributed to PE. For example, studies conducted in human pluripotent stem cells using whole‐genome sequencing (WGS) show no detectable accumulation of unintended PE‐induced mutations [77, 78]. However, extremely rare, low‐frequency clonal events remain below the detection threshold of current WGS technologies. To enhance clinical relevance, additional strategies—such as genome‐wide off‐target analysis using two‐cell embryo injection (GOTI), comparing edited and unedited lineages within the same embryo to identify low‐frequency variants—will be necessary in future studies [79].

Although later generations of prime editors such as PE2, PE3, and PE6 have been evaluated in therapeutic and experimental contexts for potential off‐target edits, most studies to date have examined only a limited number of predicted candidate sites rather than performing fully unbiased, genome‐wide profiling. These candidate‐based analyses consistently report very low off‐target frequencies, suggesting that improvements in PE efficiency have not come at the expense of specificity; however, comprehensive genome‐wide interrogation will be essential to definitively characterize off‐target landscapes across different PE generations.

## 5 Prime Editing Efficiency Prediction

The PE efficiency is significantly influenced by the design of the pegRNA and, in the case of PE3/PE5, by the design of the ngRNA used together. Because rigorous experimental optimization of pegRNA design is labor‐intensive, several computational and machine learning models have been developed to predict PE efficiency and guide optimal pegRNA selection. The first large‐scale study [80] by Kim and coworkers evaluated PE2 activity at tens of thousands of pegRNA–target pairs in human cells and identified sequence features—including RTT AND PBS lengths, GC content, and editing position—that shape PE2 performance. These data were used to build computational models that estimate pegRNA efficiency for a given target and systematically suggest RTT‐PBS length combinations, providing a foundation for sequence‐based PE design. Subsequent deep learning approaches have substantially expanded the scope and accuracy of PE prediction. Schwank and coworkers generated high‐throughput datasets covering diverse edit types and RTT architectures, and trained deep neural networks (PRIDICT and PRIDICT2.0 [81, 82]) to predict both overall PE efficiency and product purity, enabling users to rank candidate pegRNAs and select designs that maximize intended edits while minimizing undesired byproducts such as indels or unintended substitutions. Follow‐up work integrated chromatin features with reporter‐based assays to derive ePRIDICT [82], which estimates how local chromatin context modulates editing outcomes at endogenous loci and helps explain why identical pegRNAs can perform differently across genomic sites.

In parallel, Kim and coworkers [83] developed DeepPrime and DeepPrime‐FT, deep learning models trained across multiple PE systems (including different PE architectures and editing types) to predict pegRNA efficiency in a more generalizable manner. An additional model, DeepPrime‐Off, estimates off‐target PE probabilities, thereby linking pegRNA sequence features to both on‐target efficiency and potential off‐target activity.

Together, these models have transformed PE gRNA design from empirical trial‐and‐error into a data‐driven process. Integrating sequence‐based predictors with chromatin‐aware models and editor‐specific parameters (for example, PE2 versus PE6 or rPE variants) is expected to further improve the transferability of predictions to endogenous loci and complex edit types, ultimately shortening optimization cycles and accelerating the deployment of PE in both basic research and therapeutic applications.

## 6 Therapeutic Applications of Prime Editing

Most pathogenic human mutations (approximately 90%) comprise single‐nucleotide substitutions or small indels shorter than 12 bp [20]. In principle, all these mutations are amenable to correction through PE. Furthermore, PE achieves higher editing precision by avoiding the complete gene knockout typically induced by Cas9 nucleases and the unintended bystander edits associated with BEs [7, 10, 23, 84]. Consequently, researchers are increasingly applying PE in both in vivo and ex vivo contexts to correct disease‐causing mutations previously difficult to target (Table 1).

**TABLE 1:** Therapeutic Programs based on Prime Editing.

Disease	Inducing mutation	Delivery	Study name	Product name (candidate)	Phase	Company	Refs.
CGD	GT insertion to correct NCF1 GT deletion	Ex vivo (HSC)	Prime‐0101	PM‐359	Phase I/II	Prime medicine	[85, 86]
Wilson's disease	A to C substitution to correct ATP7B H1069Q or R778L mutation	In vivo (LNP)	—	—	Prescreening study	Prime medicine	[87, 88]
AATD	Edit the E342K (Pi*Z) mutation in the SERPINA1 gene	In vivo (LNP)	—	—	Lead optimization	Prime medicine	[89]

**Abbreviations**: AATD, Alpha‐1 Antitrypsin Deficiency; CGD, Chronic Granulomatous Disease; HSC, hematopoietic stem cell; IND, investigational new drug; LNP, lipid nanoparticle.

Chronic granulomatous disease (CGD) is an inherited immunodeficiency disorder in phagocytes, specialized immune cells responsible for microbial killing that fail to eliminate pathogens, leading to chronic and recurrent infections [90]. The disease is induced by dysfunctions in proteins—such as gp91phox, p47phox, p67phox, and p22phox—crucial units of the NADPH oxidase complex, an enzyme essential for producing hydrogen peroxide necessary for pathogen clearance [90]. Prime Medicine is currently developing an ex vivo PE therapy targeting a mutation (c.75_76delGT) in the *NCF1* gene, encoding the p47phox protein. This approach involves correcting the pathogenic mutation in CD34+ HSPCs derived from patients with CGD using a prime editor. Subsequently, the corrected HSPCs were transplanted into immunodeficient mice, with restored NADPH oxidase activity observed in differentiated neutrophils, leading to a reduced interferon expression [85, 91]. This research is currently undergoing evaluation in a Phase 1/2 clinical trial [86].

Wilson's disease is a life‐threatening autosomal recessive disorder characterized by defective copper metabolism, leading to toxic copper accumulation primarily in the liver and brain. This buildup causes progressive hepatic injury, neurological dysfunction, and hematologic abnormalities [92]. The condition is attributed to loss‐of‐function mutations in the *ATP7B* gene, which encodes a copper‐transporting T‐type ATPase. Ongoing gene editing research targets the most frequent pathogenic mutations, H1069Q and R778L. Prime Medicine is developing an in vivo gene‐correction strategy delivering prime editor mRNA, pegRNA, and ngRNA to hepatocytes using lipid nanoparticles (LNPs). In humanized mouse models, this approach achieved gene correction efficiencies exceeding the clinically relevant threshold and restored normal ceruloplasmin levels [87]. Building on these promising preclinical results, a prescreening study has recently been initiated to identify potential participants for the upcoming gene‐editing clinical trial [88].

PE is also being applied to the correction of repeat expansion disorders, posing significant challenges for previous gene‐editing platforms. Owing to disease severity and onset correlating with the number of repeats, these conditions cannot be effectively treated through simple base substitutions or single‐nucleotide corrections [93]. While dual Cas9 nucleases strategies have been used to excise expanded repeat segments, these methods lack the precision necessary for fine‐scale corrections. Consequently, researchers are now employing prime editors with paired pegRNAs to selectively excise pathogenic repeat expansions in disorders such as Friedreich's ataxia (FRDA), fragile X syndrome (FXS), and myotonic dystrophy type 1 (DM1) [94].

## 7 Conclusion

PE represents a transformative genome‐editing technique offering high versatility and producing highly precise, high‐purity outcomes compared to those of other targeted editing platforms. However, PE remains an evolving system with certain limitations. To address challenges such as its relatively low efficiency, researchers have made extensive efforts to optimize and refine the PE system, leading to significant improvements in efficiency over the initial version, achieved through various molecular and methodological strategies. These strategies encompass modulating cellular repair pathways (e.g., PE3, PE4/5, and PE‐SB), refining PE activity through protein engineering of the Cas protein and RT (e.g., PE2, PEmax, PEmax^**^, PE6, and rPE), and enhancing pegRNA stability using engineered pegRNA variants (e.g., epegRNA and circular pegRNA) or by exploiting protective RNA‐binding proteins (e.g., PE7). Beyond improving editing efficiency, researchers have expanded the capacity of the system for large‐scale genome modifications. These advancements include inducing secondary DNA synthesis through additional flap binding (e.g., paired‐pegRNA, TJ‐PE) and enabling gene‐length insertions by integrating the PE system with recombinases (e.g., PASTE, PASSIGE). Recent studies on the PA system show that large‐scale genomic modifications, including insertions, deletions, and replacements, are achievable without the need for recombinases, leading to higher efficiency and system simplification. These advancements enhance PE efficiency and significantly broaden its applicability, making it valuable for both therapeutic applications and fundamental biological studies.

The broad applicability of PE makes it a highly versatile platform for correcting various disease‐related mutations. However, its translation into clinical use depends on the development of efficient and cell‐type‐specific delivery systems capable of achieving effective in vivo targeting. AAV is the leading vehicle for gene therapy, but the large size of prime editors exceeds the packaging capacity of a single AAV vector. To address this, dual‐AAV systems have been utilized, employing either split‐intein architectures to reconstitute full‐length editors or, as described earlier, intein‐independent sPE strategies such as untethered RT systems [27, 30]. Alternatively, virus‐like particles (VLPs) offer a transient delivery strategy that minimizes off‐target effects by delivering the PE ribonucleoprotein complex directly, with recent engineered VLPs (eVLPs) showing improved in vivo efficacy for PE [95]. LNPs serve as a potent non‐viral delivery vehicle by encapsulating PE mRNA and pegRNAs. Several studies have reported successful in vivo PE outcomes using this approach [63, 96, 97]. However, limitations in tissue specificity and potential toxicity of LNPs remain important challenges [98]. Consequently, significant advances in delivery technology are needed to realize the full therapeutic potential of PE.

AI‐driven technologies have recently emerged as valuable tools for accelerating the optimization of genome‐editing systems. In the context of PE, AI‐based approaches have enabled systematic protein engineering and de novo protein design, as demonstrated by recent developments in tailored small binders [35] and optimized RT/nCas9 components [28]. Although these applications remain in an early stage compared with conventional molecular engineering, accumulating evidence suggests that AI‐guided design may complement experimental strategies to improve the performance and diversity of prime editors. Looking ahead, continued integration of AI‐guided design into PE development could contribute to further refinement of PE platforms, although its long‐term impact will depend on experimental validation across diverse cell types and delivery settings. Ultimately, if future technological innovations continue to converge across molecular engineering, delivery strategies, and AI‐guided design, these advances may enable the field to move closer to permanent cures for genetic diseases, a longstanding goal in biomedical science.

## Conflicts of Interest

The authors declare no conflicts of interest.

## Data Availability

The authors have nothing to report.
